# Genome Sequence Analysis of *Auricularia heimuer* Combined with Genetic Linkage Map

**DOI:** 10.3390/jof6010037

**Published:** 2020-03-16

**Authors:** Ming Fang, Xiaoe Wang, Ying Chen, Peng Wang, Lixin Lu, Jia Lu, Fangjie Yao, Youmin Zhang

**Affiliations:** 1 Lab of genetic breeding of edible mushromm, Horticultural, College of Horticulture, Jilin Agricultural University, Changchun 130118, China; fangming@jlau.edu.cn; 2 Engineering Research Centre of Chinese Ministry of Education for Edible and Medicinal Fungi, Jilin Agricultural University, Changchun 130118, China

**Keywords:** *Auricularia heimuer*, Alcohol dehydrogenase, CAZymes, genome, linkage map, mating-type, phylogeny

## Abstract

*Auricularia heimuer* is one of the most popular edible fungi in China. In this study, the whole genome of *A. heimuer* was sequenced on the Illumina HiSeq X system and compared with other mushrooms genomes. As a wood-rotting fungus, a total of 509 carbohydrate-active enzymes (CAZymes) were annotated in order to explore its potential capabilities on wood degradation. The glycoside hydrolases (GH) family genes in the *A. heimuer* genome were more abundant than the genes in the other 11 mushrooms genomes. The *A. heimuer* genome contained 102 genes encoding class III, IV, and V ethanol dehydrogenases. Evolutionary analysis based on 562 orthologous single-copy genes from 15 mushrooms showed that *Auricularia* formed an early independent branch of Agaricomycetes. The mating-type locus of *A. heimuer* was located on linkage group 8 by genetic linkage analysis. By combining the genome sequence analysis with the genetic linkage map, the mating-type locus of *A. heimuer* was located on scaffold45 and consisted of two subunits, α and β. Each subunit consisted of a pair of homeodomain mating-type protein genes *HD1* and *HD2*. The mapping revealed conserved synteny at the whole mating-type loci and mirror symmetry relations near the β subunit between *A. heimuer* and *Exidia glandulosa*. This study proposed the potential for the bioethanol production by consolidated bioprocessing of *A. heimuer*. It will promote understanding of the lignocellulose degradation system and facilitate more efficient conversion of the agricultural wastes used for mushroom cultivation. It also will advance the research on the fruiting body development and evolution of *A. heimuer*.

## 1. Introduction

*Auricularia heimuer* is one of the most popular edible fungi in China and has high economic and medicinal value [1]. The fruiting body of wood ear is earlike and rich in gelatin. Its cultivation history in China can be traced back more than 1100 years. The traditional cut-log cultivation method of *A. heimuer* was described in Compendium of Materia Medica, which was published in 1578 by Li Shizhen [2]. The modern cultivation method of *A. heimuer* is mushroom bag cultivation (cultivation with intermittent mist under sunlight) [3], which has made it possible for the formation of huge industrial chains.

Under wild conditions, *A. heimuer* grows mostly on fallen trees and stumps (Vilela et al. 1982), indicating that *A. heimuer* is a wood-rotting fungus [4]. The sawdust of broad-leaved trees rich in lignin is often used for wood ear cultivation. With the increasing awareness of eco-environmental protection, the exploitation of forest resources has been restricted in China, resulting in a decrease in the supply of sawdust and an increase in the cost of mushroom cultivation. A large amount of crop straw is burned every year in China because of its inefficient utilization, and this causes serious air pollution. Compared with sawdust, crop straw is rich in cellulose and hemicellulose, which is not optimum for the cultivation of wood rot mushrooms [5]. However, sawdust resources are limited, research into alternative resources has gradually increased, and the ability of *A. heimuer* to degrade lignocellulose is still not clear at a molecular level. Energy security is becoming an important worldwide topic and bioethanol is one of the renewable resources that could help to ensure energy security. The edible fungi *Flammulina velutipes* [6], *Pleurotus ostreatus*, *Tricholoma matsutake*, and *Agaricus blazei* [7] have been reported to have the ability to convert lignocellulose into bioethanol, but similar studies on *A. heimuer* have not been reported so far. *Auricularia* belongs to the family Agaricomycetes, but is different from other mushrooms in this family [8]. In *Auricularia* there is no differentiation of stalks and caps, indicating that *Auricularia* is evolutionarily distant from other species in Agaricomycetes. Traditionally, molecular evolutionary studies have used phylogenetic trees that were constructed of one or several molecular markers, so the results may have some limitations. High-throughput sequencing technologies have been used to sequence the genomes of important edible fungi [9,10] and model fungi [11,12] in Agaricomycetes. The availability of these sequences has made it possible to study *A. heimuer* at the genomic level. Sexual reproduction of *A. heimuer* is an important part of its sexual life cycle, genetics, and breeding. Classical genetics has shown that the mating system of *A. heimuer* is bipolar heterothallism [13], but little is known about the structure of the locus and coding sequences of this mating-type.

To further explore the characteristics of *A. heimuer*, the whole genome sequence of *A. heimuer* was obtained by next-generation high-throughput sequencing. The potential of *A. heimuer* for lignocellulose decomposition and ethanol conversion was investigated by analyzing related genes. A phylogenomic tree was constructed of single-copy orthologous genes in the genome. The structure of the mating-type locus was analyzed. The coding sequences of mating-type genes were determined.

## 2. Materials and Methods

### 2.1. Strains and Culture Conditions

Monokaryotic *A. heimuer* strains A14-8 and A14-5 came from dikaryotic strain A14 (Qihei No. 1), which originated from a wild strain collected in AnTu county, Jilin province. Strain A18-119 came from dikaryotic strain A18 (Qihei No. 2) [14]. All strains were preserved in the College of Horticulture, Jilin Agricultural University. Strain A14-8 was used for the de novo genome sequencing and strains A14-5 and A18-119 were used for the re-sequencing. The genome sequence of *A. heimuer* strain Dai 13782 was obtained from GenBank (accession number NEKD00000000.1). The three monokaryotic strains A14-8, A14-5, and A18-119 were isolated from *A. heimuer* fruiting bodies by single-spore isolation. The strains were inoculated onto potato dextrose agar medium in 90-mm Petri dishes and cultured for 7 days at 25 °C. Five mycelial blocks of 0.5 mm^3^ were inoculated into agar-free potato dextrose liquid medium and cultured in shakers at 120 rpm for 7 days in the dark. F1 generations were obtained by crossing A14-5 and A18-119. The monokaryotic strains were selected from the F1 generation and used as the mapping population to construct a genetic linkage map [14].

### 2.2. Genomic DNA Extraction

Genomic DNA was extracted from strains A14-8, A14-5, and A18-119 using a QIAamp DNA Mini Kit (Qiagen, Dusseldorf, Germany). The quality of the extracted genomic DNA was determined using a NanoDrop 2000 UV-Vis spectrophotometer (Thermo Scientific, Waltham, MA USA) and Qubit 2.0 fluorometer (Life Technologies, Waltham, MA USA).

### 2.3. DNA Library Construction and Illumina Deep Sequencing

Two genome sequencing libraries with 500 bp and 2 kb of A14-8 were constructed for de novo sequencing using the NEBNext Ultra DNA Library Prep Kit for Illumina (E7370L, NEB, Ipswich, MA, USA) following the manufacturer’s instructions. Paired-end (2 × 150 bp) sequencing of the DNA libraries was performed on the Illumina HiSeq X system (Illumina, USA).

### 2.4. Gene Assembly, Prediction, and Functional Annotation

The clean sequence reads were obtained by filtering the raw data to remove reads that contained adapters The reads were assembled using the CLC Genomics Workbench (version 6.5) with the default parameters. Genes were predicted using GeneMark version 4.3 [15], Fgenesh [16], and Glimmer HMM (version 3.0) [17]. GeneWise [18] was used to predict the gene structure through searches against similar protein sequences in the UniProt database. Finally, EVidenceModeler was used to integrate the gene predictions to obtain the final set of predicted genes [19,20]. For functional annotation of the predicted genes, the genes were compared using BLAST (version 2.7.1) with the Kyoto Encyclopedia of Genes and Genomes (KEGG; http://www.genome.jp/kegg/) protein databases. The E-value cutoff for BLAST comparison was 0.001. The protein domains were identified using InterProScan [21]. Lineage-specific gene expansion and contraction were estimated using the CAFÉ software.

### 2.5. Annotation of Genes Encoding Carbohydrate-Active Enzymes (CAZymes)

The annotated *A. heimuer* genome and related genomes from GenBank (Table S1) were analyzed using the CAZy annotation pipeline [22]. Searches against the Pfam protein families’ database were performed to functionally annotate carbohydrate-active modules in the annotated genes, namely glycoside hydrolases (GHs), glycosyltransferases (GTs), polysaccharide lyases (PLs), and carbohydrate esterases (CEs). Auxiliary activity (AA) modules were identified using dbCAN2 [23].

### 2.6. Phylogenomic Analysis of A. heimuer and Related Genomes

Single-copy genes in OrthoDB 7 [24] were aligned using MAFFT version 6.7 [25] with the “genafpair” option and default parameters. A hidden Markov model (HMM) was constructed for each gene family using HMMER version 3.0 [26]. Orthologous gene families from 16 mushroom genomes *A. heimuer*, *Tremella fuciformis*, *Laccaria bicolor*, *A. subglabra*, *Lentinula edodes*, *Coprinus cinereus*, *F. velutipes*, *Exidia glandulosa*, *P. ostreatus*, *Serpula lacrymans*, *Schizophyllum commune*, *Agaricus bisporus*, *Morchella conica*, *Volvariella volvacea*, *Ganoderma lucidum*, and *Tremella mesenterica* (Table S1) were selected using the HMMs of the genes with E values < 1 × 10^−50^ and the highest HMM scores. Conserved regions were detected using Gblocks version 0.91b [27]. ProtTest version 3.2 [28] was used to select the appropriate amino acid replacement models with the maximum likelihood method. The phylogenomic tree was built using RAxML version 8 [29].

### 2.7. Analysis of Gene Families Expansion and Contraction

The program of CAFÉ 4.2.1 was used to estimate the CAZymes gene families expansion and contraction [30]. The birth and death (λ) rates were estimated by using the program lambda with the -s option. Two more parameters were set as the P value threshold (0.05), and the number of random samples used the default value (1000) following the Monte Carlo re-sampling procedure.

### 2.8. Preparation of Mating-type Mapping Population and Mapping

Single sequence repeat (SSR) markers for the linkage map were developed according to the whole genome sequence (GenBank NCVV00000000), which was sequenced earlier by our research team [14]. One of the monokaryotic strains from the F1 of 119-5 was selected randomly from the mapping population for pairwise mating with the other strains. The mating-type mapping population was determined by identifying clamp cell connections under an optical microscope. The mapping population was separated into two groups, one that shared mating compatibility with Gx and one that did not. JoinMap version 4.0 [31] was used for the linkage analysis of the mating-type locus. The mating-type locus was mapped onto the *A. heimuer* genetic linkage map, named MAT-A. The genetic linkage map of *A. heimuer* was constructed as described previously [14].

### 2.9. Analysis of Genomic Structure of the Mating-type Locus in A. heimuer

The nucleotide sequences of the homeodomain mating-type protein genes (HD1 and HD2), mitochondrial intermediate peptidase gene (mip), and the beta-flanking genes (beta-fg) *C. cinereus* (X79687.1, X79688.1, AF126786.1), *S. commune* (X77949.1), *Pleurotus djamor* (AY462112.1, AY462110.1), *Pholiota nameko* (AB435542.1), and *F. velutipes* (HQ630588.1, HQ630589.1) were used as query sequences. The obtained genomic sequences were aligned by blastp and tblastn with E-values < 1 × 10^−50^. Fgenesh was used to predict genes that were not detected using blastp and tblastn.

### 2.10. Gene Synteny Analysis of MAT-A of A. heimuer, A. subglabra, and E. glandulosa

The gene synteny of MAT-A of *A. heimuer*, *A. subglabra*, and *E. glandulosa* was analyzed using the MCScanX toolkit [32] with the default parameters. The gene synteny map was drawn using MCScanX [32].

## 3. Results

### 3.1. The Genome Features of A14-8 

The obtained A14-8 genome sequence was 43.6 Mb in length (NCVV01000001) with 57% GC content. All the sequence data were assembled into 535 scaffolds with a N50 of 269,985 bp. The longest scaffold was 964,551 bp long. The assembled genome was predicted to contain 15,997 genes by combining the results from AUGUSTUS [33] and Fgenesh [34,35]. The average length of the *A. heimuer* genes was 1946 bp, and the average length of the coding sequences was 1588 bp including exons (average 270 bp) and introns (average 73 bp) (Table 1). The results of assignment against protein databases such as KEGG, InterPro signature, signal peptide, transmembrane domain and pfam alignment are shown in Tables S2–S5.

### 3.2. Lignocellulolysis 

We detected 509 CAZymes in the A14-8 genome by searching the CAZy database (Table S3) [22], including 60 carbohydrate-binding modules (CBMs), 18 CEs, 265 GHs, 51 GTs, and 11 PLs. The A14-8 genome had the largest number of CAZyme coding genes among the 13 fungi genomes that were analyzed (Table 2). Further, the number of CAZyme coding genes in the A14-8 genome was 1.56 times higher than the average number of these genes in the eight wood-rotting fungi (*T. fuciformis*, *L. edodes*, *F. velutipes*, *P. ostreatus*, *S. commune*, *G. lucidum*, *S. lacrymans*, and *T. mesenterica*) and 1.36 times higher than the average number of these genes in the three straw-rotting fungi (*A. bisporus*, *V. volvacea*, and *C. cinereus*). The A14-8 genome also had more genes encoding GHs (265) and CBMs (60) than all the other genomes except the *C. cinereus* and *V. volvacea* genomes (Table 2). These results showed that *A. heimuer* has a large amount of candidate genes related to cellulose and hemicellulose degradation, as reflected mainly by the hydrolysis and oxidative reactions [36]. 

Floudas [37] identified 17 CAZyme families involved in wood degradation. Twelve of these families comprising 109 genes were found in *A. heimuer* (Table 3); the genes encoding GH74, CE1, CE12, CE15, and CE16 were not detected. The abundance of crystalline cellulose is an important marker for identifying white rot fungi [10]. Genes encoding key enzymes that reflect the nutrient utilization characteristics of *A. heimuer* were also detected, including 2, 5, 43, and 19 genes that encode members of the GH6, GH7, CBM1, and A9 subfamilies, respectively. These numbers were 29, 30, 40, and 30 higher than the numbers of the corresponding genes of the wood-rotting fungi *L. edodes*, *F. velutipes*, *S. commune*, and *G. lucidum*, and 40 higher than for the straw-rotting fungus *A. bisporus* (Table 3). In terms of the total gene numbers in wood-rotting fungi that are related to crystalline cellulose degradation, *P. ostreatus* has the largest number, then *A. subglabra* and *A. heimuer*. *V. volvacea* and *C. cinereus* have the largest gene numbers among all the wood-rotting and straw-rotting fungi. The results are consistent with the strong ability of straw-rotting fungi on the crystalline cellulose degradation. 

As a contiguous amino acid sequence within a carbohydrate-active enzyme, CBMs have carbohydrate-binding activity. According to the results of Table 3, genes numbers of CBM1 accounted for 71.7% of total CBM genes. All identified CMB1 in *A. heimuer* had cellulose-binding modules. The length of CBM in the *A. heimuer* genome were all 29 amino acid in length according to the results of alignment (Table S6). Five GH7 genes and two GH6 genes were identified. It was found that six of these genes had cellulose-binding modules combined with glycoside hydrolase family (Table S6). Nineteen genes encoding members of the AA9 family were identified in the *A. heimuer* genome (Table S7). They accounted for 18.3% of the total number of genes encoding AA enzymes. AA9 (formerly GH61) proteins are copper-dependent lytic polysaccharide monooxygenases (LPMOs) that are an important oxidative enzyme for the decomposition of polysaccharides such as cellulose [36]. There were six and four more AA9 genes in the *A. heimuer* genome than there were in the *L. edodes* and *G. lucidum* genomes, respectively. The two possible ways of polysaccharide decomposition, hydrolysis and oxidation, indicate the diversity and efficiency of this process in *A. heimuer*.

*A. heimuer* is a wood-rotting fungus. The *A. heimuer* genome contained 104 genes that encode members of the AA families including AA1–AA9 and AA12 (Table S7). This number of genes is more than the numbers of corresponding gene in the *F. velutipes*, *S. commune*, and *G. lucidum* genomes. The AA1 enzymes are multicopper oxidases that use diphenols as electron donors and oxygen as the acceptor, and are one of the most important CAZyme families that degrade lignin. One ferroxidase (AA1_2)-encoding gene and four laccase-like multicopper oxidase (AA1_2 and AA1_3) encoding genes were found in the *A. heimuer* genome. The organization of genes in the *A. heimuer* genome that encodes AA enzymes is similar to that found in ascomycetes [38]. Class II lignin-modifying peroxidases are also important CAZymes for lignin degradation and they are used as markers to discriminate white rot fungi and brown rot fungi. A total of 21 AA2 subfamilies-encoding genes were identified in the *A. heimuer* genome. Twelve genes were identified as cytochrome-c peroxidase, and five genes were identified as 1,3 exoglucanase based on the annotation of funcat. Eleven genes were identified as peroxidase MNP2, one gene was identified as cytochrome c, one gene was identified as peroxidase, three genes were identified as cytochrome c peroxidase, three genes were identified as generic peroxidase, and one gene was identified as ascorbate oxidase (Table S8). The total number of AA2 subfamilies genes was 2.1 times more than in the *G. lucidum* and *L. edodes* genomes and 2.3 times more than in the *P. ostreatus* genome. The results for the genes that encode members of theAA1 and AA2 subfamilies indicates that lignin degradation by *A. heimuer* depends mainly on the aiding of AA2 subfamilies.

Thirty genes that encode members of the AA3 family were detected, which is the largest number detected among the AA families. Among the 30 genes, at least 22 genes were AA3-2 family genes that encode the aryl-alcohol oxidases and glucose 1-oxidases that are required for AA2 family genes to oxidize lignin [38], eight of which can form multi-modular structures with enzymes in the AA8 family. The large number of genes encoding AA2 and AA3-2 family enzymes forms a solid foundation for efficient lignin degradation in *A. heimuer*. Genes encoding three AA12 enzymes with pyrroloquinoline quinone-dependent reductase activity also have been found in the *A. heimuer* genome [39], but not in the *L. edodes*, *S. lacrymans*, or *G. lucidum* genomes, and only one copy was found in *P. ostreatus*.

### 3.3. Gene Families Expansion and Contraction of CAZymes

**Figure 1:**
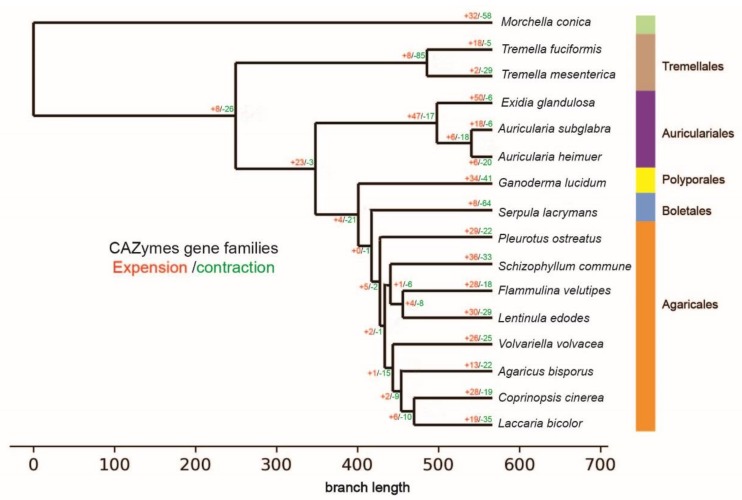
The expansions and contractions of the CAZyme gene families. Numbers associated with each branch designate the numbers of gene families that have expanded (red) or contracted (green) since the split from the common ancestor. The color bars correspond to different orders.

From the perspective of different classes, the expansions and contractions of CAZyme families are different in evolution (Figure 1). Compared with the species of Agaricomycetes, Tremellomycetes species (*T. fuciformis* and *T. mesenterica*) went through a more dramatic change in gene families, losing 85 gene families and gaining eight. In Agaricomycetes (including the species of Auriculariales, Polyporales, Boletales, and Agaricales), the Auricularia evolved first (Figure 1). According to the results of expansions and contracts, Agaricales obtained more gene families (47) and lost fewer gene families, while other species belonging to different orders obtained four gene families and lost 17 as a single branch.

Analysis of changes in gene families of CAZymes revealed six expended gene families and 20 contracted gene families in the *A. heimuer* genome (Figure 1). The contracted gene families included five AA families, 12 GH families, two CBM families and one GT family, three of which were identified as rapidly evolving families. The expended gene families included four GH families and two CBM families, and no rapidly evolving families were found (Table S9). According to the results, the AA3 gene family was the contraction family found the fastest (*p* value = 5.93 × 10^−5^), and the number of contraction genes was eight (Table S9). In the previous analysis, it was demonstrated that AA3 was the family with the largest number (30 genes). Among the 30 genes, only three AA3-3 genes, one AA3-4 gene and zero AA3-1 genes were identified. AA3-2 genes accounted for 66.7%. Based on the previous analysis in this research, lignin degradation by *A. heimuer* depends mainly on the aiding of AA2 famliy genes. It is known that AA3-2 family genes encode the aryl-alcohol oxidases and glucose 1-oxidases that are required for AA2 famliy genes to oxidize lignin. Although the AA3 family has been under contraction, a large amount of AA3-2 genes was left and performed the function of lignin degradation with AA2 famliy genes. The other family genes in AA3 having been lost in the evolution. These observations indicate that even the AA3 family was subject to contraction evolution, with *A. heimuer* retaining the largest number of AA3-2 famliy genes. This might have been due to better adaption to utilize lignin in the process of evolution.

### 3.4. Alcohol Dehydrogenases

**Figure 2:**
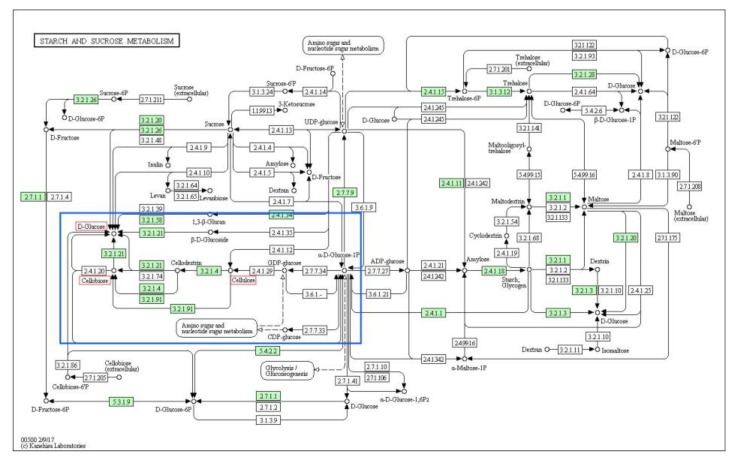
The cellulose degradation pathway of *A. heimuer*. The red and green boxes indicate the substrates and existing homologous genes of the enzyme, respectively. The white boxes mean the enzymes that were not identified in *A.heimuer* genome. The blue box indicates the core region of the function. The figure was generated by a KEGG mapper.

**Figure 3:**
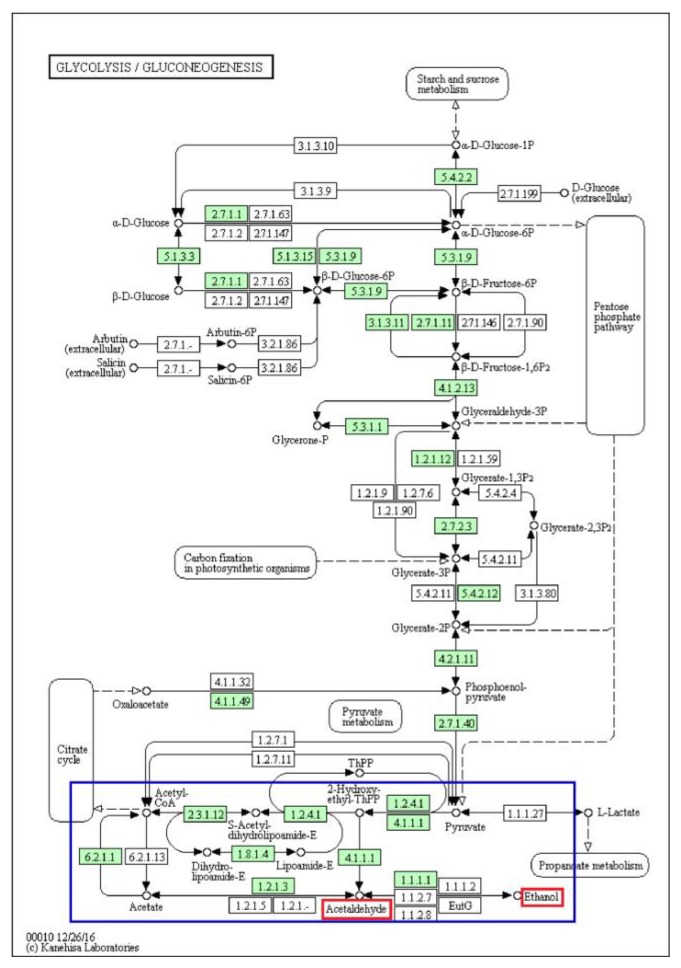
Pathway of the biosynthesis of ethanol. The red and green boxes indicate the substrates and existing homologous genes of the enzyme, respectively; The white boxes mean the enzymes that were not identified in *A.heimuer* genome. The blue box indicates the core region of the function. The figure was generated by a KEGG mapper.

Alcohol dehydrogenases (ADHs) are a large family of enzymes that are in charge of the reversible oxidation of alcohols to aldehydes through the concomitant reduction of NAD1 or NADP1 [40]. A total of 102 genes that encode class III, IV, and V ethanol dehydrogenases were identified in the *A. heimuer* genome, which was 1.76 times more than the corresponding genes (58) in the *F. velutipes* genome [41]. This results indicate that *A. heimuer* had the potential to synthesize bioethanol by converting lignocellulose to polysaccharides, then to sugars, and finally to ethanol. (Tables S10 and S11). It is known that cellulose can be degraded to cellobiose then glucose by fungi [42]. The glucose can be converted to pyruvate via the Embden–Meyerhof–Parnas (EMP) pathway [7], then be converted to acetaldehyde and, finally, ethanol for the function of ADHs. According to the annotation of KEGG pathway, we checked *A. heimuer* genes during the conversion from cellulose to ethanol (Figure 2 and Figure 3), which showed that acetaldehyde can be conversed to ethanol for the function of ADHs (EC 1.1.1.1).

### 3.5. Phylogenomic Analysis

**Figure 4:**
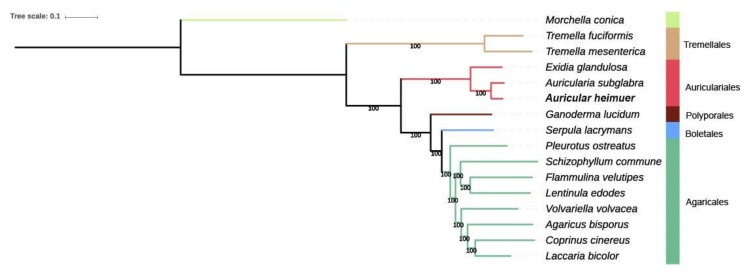
Phylogenomic tree of 15 mushroom species based on single-copy orthologous genes. The mushroom species separated into three main groups. *Morchella conica* was used as the outgroup. The different orders are indicted by different colored bars. Tree scale = 0.1. The numbers under bars indicate 100% bootstrap.

Phylogenomic analysis was carried out using the published sequences of 562 single-copy orthologous genes of 15 mushroom species belonging to orders Auriculariales, Boletales, Polyporales, Agaricales, and Tremellales (Table S1). The orthologous genes of *M. conica* were used as the outgroup. The species in Agaricales and Tremellales were separated into two clades. The two *Auricularia* species formed an earlier independent branch in the Agaricomycetes clade (Figure 4). Other mushrooms that showed differentiation of stipe and pileus formed a separate branch, which is consistent with the morphological classification. 

### 3.6. Mating-Type Analysis of Mapping Population

One-hundred and thirty-eight strains were identified from F_1_ 119-5 as monokaryotic strains due to the presence of a clamp connection under an optical microscope. One of the 138 strains was designated G1 and selected to mate with the remaining 137 strains in the mapping population. The compatible mating-types with G1 were designated as A2. A total of 72 compatible mating reactions and 65 incompatible mating reactions were detected, indicating the mating-type of 72 strains was A1 and the mating-type of 65 strains was A2 (Table S12). 

### 3.7. Mating-Type Location on the Genetic Linkage Map

**Figure 5:**
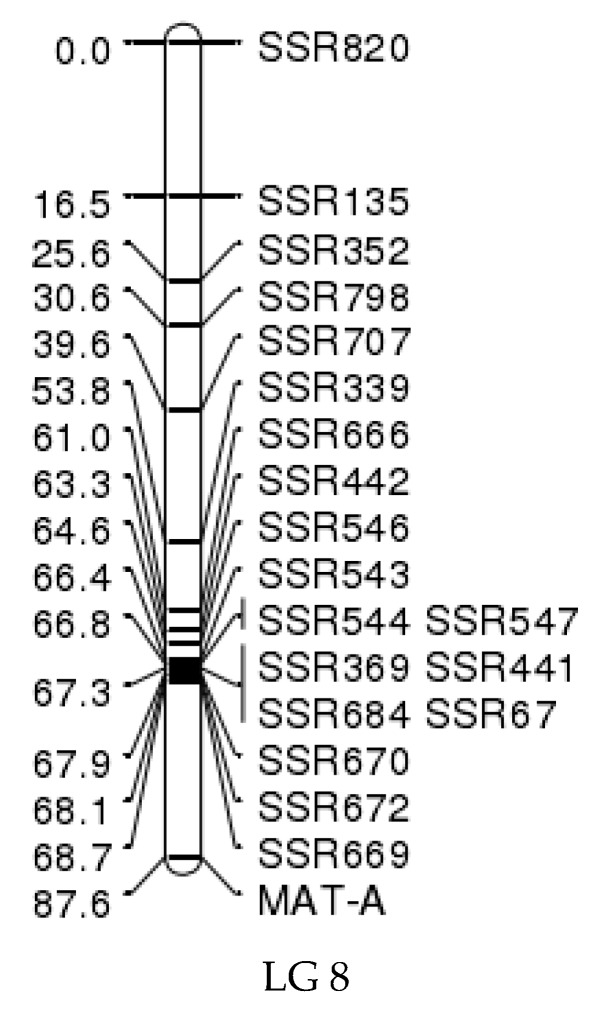
MAT-A mapping on the genetic linkage map. The bar in the middle represents the linkage group, the number on the left represents the genetic distance, the different SSR markers used for the construction of linkage map in this research are listed on the right according to the linkage relation. MAT-A represents the mating-type loci.

The results obtained using JoinMap version 4.0 showed that mating-type strains were located at the locus at 87.6 cM on linkage group 8 (Figure 5) [14]. This indicates that the simple sequence repeat (SSR) molecular marker SSR669 (scaffold45_size276651_(CTG)6_18_209253_209270) was linked to the mating-type locus (MAT-A), which was 18.9 cM distant from the mating-type locus (Figure 5).

### 3.8. Structure of the Mating-type Locus

The blastp and tblastn alignments indicated that the mating-type genes of strain A14-8 were on scaffold45, which is consistent with the linkage map positioning results. The SSR marker SSR669 that was linked closely to MAT-A was also located on scaffold45. SSR669 was located downstream of *HD*148-2-1 (GenBank: MN267021) at a distance of 88.535 kb. There were two pairs of homeodomain mating-type protein genes *HD*148-1-1 (GenBank: MN267022) and *HD*148-2-1, *HD*148-1-2 (GenBank: MN267023), and *HD*148-2-2 (GenBank: MN267024) on scaffold45. *HD*148-1-1 and *HD*148-2-1 encoded α subunits of 683 and 693 amino acids, respectively, and the distance between the two genes was 153 bp. *HD*148-1-2 and *HD*148-2-2 encoded β subunits of 626 and 628 amino acids, respectively. The distance between the two genes was 294 bp. There was 36.725 kb between the genes encoding the α and β subunits (Table 4).

**Figure 6:**
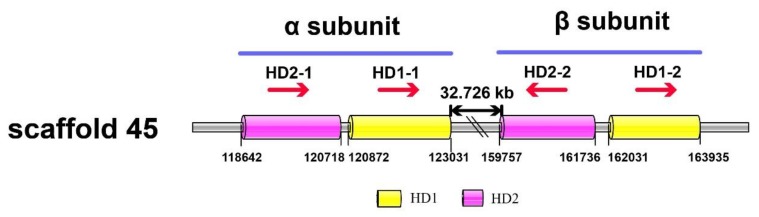
The mating-type structure of 14-8. The blue lines indicate the mating-type locus, the red arrows indicate the transcriptional direction of the genes, and the gray bar represents the region of scaffold45 that contains the mating-type genes. The numbers below are the positions of the two ends of the mating-type genes on scaffold45. The gap between the two subunits is 32.726 kb.

The results for strains A14-5, 18-119, and Dai 13782 show that the mating-type locus of *A. heimuer* contained two pairs of genes that encoded two subunits of the homeodomain proteins. The *HD*1 genes varied in length from 593 to 715 bp, and the *HD*2 genes varied from 587 to 650 bp. The distance between genes encoding the α and β subunits varied in length from 36 to 41 kb (Table 4 and Figure 6).

The genes encoding mitochondrial intermediate peptidase (*mip*) and *beta-fg* were linked closely to the mating-type locus in A14-8, which is similar to what was found for other edible mushrooms [43]. The genes 148*mip* (GenBank: MN267034) and 148*beta-fg* (GenBank: MN267037) were on scaffold45 and encoded proteins of 767 and 231 amino acids, respectively. But unlike in other fungi genomes, in the *A. heimuer* genomes these two genes were on the one side of the mating-type locus and not flanking it (Figure 6). The 148*beta-fg* gene was downstream of *HD148-1-2* at a distance of 51.913 kb and the 148*mip* was downstream of *beta-fg* at a distance of 6.32 kb (Table S13).

### 3.9. Analysis of the Synteny of MAT-A in the A. heimuer, A. subglabra, and E. glandulosa Genomes

**Figure 7:**
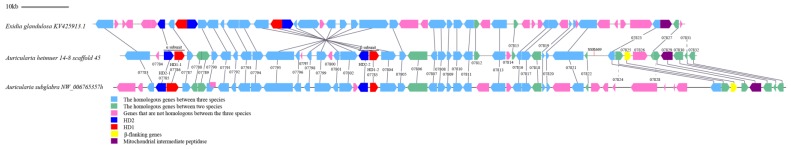
Synteny of genes on MAT-A of A. heimuer, A. subglabra, and E. glandulosa.

MAT-A of the *A. heimuer* strain A14-8 was on scaffold45 at the locus from positions 110,367 to 230,620 bp, a total of about 12 kb. The mating-type genes *HD1-2* and *HD1-1* that encode α subunits were located upstream of the MAT-A locus, and the mating-type genes *HD*2-2 and *HD*2-1 that encode β subunits were in the middle of the MAT-A locus. The results show that the genes in the MAT-A locus were highly conserved between *A. heimuer* and *A. subglabra* (Figure 7).

A total of 51 genes were detected in the MAT-A locus of *A. heimuer* and 43 (84.3%) of them were homologous to *A. subglabra* genes. Both *mip* and *beta-fg* were found downstream of MAT-A. There was conserved synteny between the homologous genes in the MAT-A locus of *A. heimuer* and *A. subglabra*, indicating the synteny in this locus were highly conserved. A total of 33 (64.71%) homologous genes were detected in the MAT-A locus of *E. glandulosa*. The mating-type genes flanking the β subunit showed mirrored symmetry, indicating the genes at this site were reversed during evolution (Figure 7). Therefore, the number of homologous genes and conserved synteny indicate that the homology between *A. heimuer* and *A. subglabra* was higher than it was between *A. heimuer* and *E. glandulosa*, which is consistent with the phylogenomic relationship of the three species.

Different color bars represent different genes, and their direction represents the direction of gene transcription. Light blue stripes represent homologous genes shared by three species, green stripes represent homologous genes shared by two species, and pink stripes represent non-homologous genes between the three species. Homologous genes are linked by black lines, dark blue stripes represent the HD1 gene, red stripes represent the HD2 gene, and subunits re marked by black lines above them. The purple strip represents the gene encoding mitochondrial intermediate peptidase. Numbers represent the number of different genes on MAT-A, and SSR669 represents the closest SSR marker to the mating-type locus on linkage map.

## 4. Discussion

### 4.1. Genome Features

The strain A14-8 used in this study was from the cultivar A14 that originated from a wild strain collected in AnTu county, Jilin Province. The strain Dai 13782 was from a wild strain found in Heilongjiang Province. Both Heilongjiang and Jilin Province are the two major production areas of these strains, so the two genomes can be considered as representative of different major production areas in China. The size of the A14-8 genome was 43.6 Mb, which was about 6 Mb shorter than the Dai 13782, and the scaffold numbers of A14-8 were four times more than the numbers of Dai 13782. The main reason for this should be lying in the different sequencing methods.

### 4.2. CAZymes

The Pfam database was used to identify the CAZymes and 120 more genes were identified in this study compared with the number reported by Yuan et al. [1]. The result of Dai 13782 was based on a comparison of BLASTP against the CAZy database (http://www.cazy.org/). The results of B14-8 were based on dbCAN2, a toolkit developed for CAZymes combined with Pfam. The differences between the two strain results could be mainly because of annotation pipelines. The correspondence between CAZy families and those in PFAM/INTERPRO or DBCAN is far from perfect [22]. In other words, the different results provided an alternative reference to exploit CAZymes and supply more candidates for further study. Genes encoding laccase-like multicopper oxidases (AA1_3) and ferroxidases (AA1_2) were found in *A. heimuer* and *A. subglabra* genomes, suggesting these genes may be common in the Auriculariaceae family. These results are very similar to those obtained for ascomycetes and support the speculation that genes encoding AA1_3 enzymes may have originated from the common ancestor of basidiomycetes and ascomycetes, or by the horizontal transfer of genes between species [38]. Quantitative differences between the AA1 and AA2 families indicates there were more genes encoding AA1 enzymes than AA2 enzymes in the genomes of *L*. *bicolor, L*. *edodes, C*. *cinereus, F*. *velutipes, P*. *ostreatus, S*. *lacrymans, A*. *bisporus, M*. *conica, V*. *volvacea, G*. *lucidum*, and *T*. *mesenterica*. However, there were 4.2 times more genes encoding AA2 enzymes than AA1 enzymes in the *A. heimuer* genome, indicating that the AA2 famliy genes play crucial roles in the lignin degradation ability of *A. heimuer* [38]. According to the ability of wood rot fungi to degrade lignin in plant cell walls, they can be classified as white rot fungi or brown rot fungi. The main differences between these two nutritional types were the higher presence of the PODs and the larger number of enzymes that act on crystalline cellulose in white rot fungi compared with brown rot fungi [10]. Comparative genomics analysis revealed the existence of intermediate types between white rot fungi and brown rot fungi, such as *Botryobasidium botryosum* and *Jaapia argillacea*. *A. heimuer* was identified as a typical white rot fungus in this study because of the presence of PODs and the large number of enzymes that act on crystalline cellulose. Moreover, the genes that encode enzymes in the A2, CBM1, GH6, GH7, and AA9 families are important for the lignocellulose degradation of *A. heimuer* and can be considered as core candidate genes for the nutritional utilization and development of molecular markers for breeding high-yield cultivars of *A. heimuer*. To efficiently utilize these genes in future studies, the transcriptomes of *A. heimuer* should to be analyzed at different developmental stages to detect the most active lignocellulose degrading genes.

### 4.3. Expansion and Contraction of CAZymes

Based on the analysis of CAZyme gene family expansion and contraction, we found that the number of contracted gene families (20) in *A. heimuer* were significantly more than the number of expanding gene families (6) in its evolution. Twenty-five of them were related to lignocellulose degradation, while only one gene family was involved in the synthesis of cell wall. This suggests that *A. heimuer* enhanced its nutrition utilization ability by changing the composition and quantity of genes related to lignocellulose degradation during its evolution. The most rapidly evolving family was AA3, with eight genes contracted. It seemed that this kind of evolutionary process is not beneficial to the nutritional utilization of *A. heimuer*, but through statistical analysis most of the AA3-2 family genes are kept in its evolutionary process. Its function is to participate in the degradation of lignin with AA2 family genes, and the number of these two gene families accounts for 49% of the total AA families’. In addition, the gene families related to starch degradation contracted in *A. heimuer* included CBM20, GH13, GH15, and GH27. However, these families are very rich in *L. edodes* [44]. This shows that different species may take different evolutionary approaches to nutrition utilization. Based on the above analysis, it is speculated that *A. heimuer* has economic and effective ways of improving lignin utilization in evolution.

### 4.4. Alcohol Dehydrogenases

Alcohol dehydrogenases can be used for the large-scale production of bioethanol, and this process has been studied extensively in *Saccharomyces cerevisiae* [40]. Mushrooms can convert cellulose into bioethanol because ethanol dehydrogenases can catalyze cellulose to acetaldehyde then ethanol. This has been identified in *F. velutipes*, *P. ostreatus*, and *Agaricus blazei*, and has been used in the production of alcoholic beverages [7]. The *F. velutipes* genome showed a strong potential for alcohol conversion, encoding 58 homologs to alcohol dehydrogenase genes covering a broad range of possible substrates. A total of 102 genes that encode alcohol dehydrogenases were identified in the *A. heimuer* genome, which was 1.76 times more than was identified in the *F. velutipes* genome. Further, a large number of genes that could decompose lignocellulose were found in the *A. heimuer* genome. The presence in *A. heimuer* of the consolidated bioprocessing steps from cellulose to bioethanol, including cellulose conversion to polysaccharides, hydrolysis of polysaccharides to sugars, and fermentation of sugars to alcohol, indicates that *A. heimuer* has the potential to synthesize bioethanol. According to a study of Tokumitsu et al., alcohol fermentation by *Agaricus blazei* depends on both the EMP pathway and ED pathway [7]. Based on analysis of the KEGG pathway in this study, only genes involved in EMP were identified, and no genes were found in the Entner–Doudoroff pathway. Until now, there have only been a few studies on alcohol dehydrogenases and none on the production of bioethanol in *A. heimuer*. To clarify which genes are involved in ethanol production, further studies on the ethanol dehydrogenases in the *A. heimuer* transcriptome and the expression of ethanol dehydrogenases in different tissues and at different developmental stages are required to evaluate the feasibility of bioethanol production by *A. heimuer*.

### 4.5. Phylogenomic Analysis

The results by phylogenomic analysis showed that *A. heimuer*, *A. subglabra*, and *E. glandulosa* formed an *Auricularia*-related clade, which indicates that *Auricularia* differentiated earlier than the other genera in Agaricomycetes. This conclusion can also be drawn from the morphological differences of the fruiting body between *A. heimuer* and other genera in Agaricomycetes. Wood-rotting and straw-rotting fungi clustered into two groups based on their nutritional types. In this study, 562 single-copy orthologous genes were used in the evolutionary analysis. This is more than the genes number used to analyze the phylogenies of *V. volvacea* [45], G. lucidum [46], and 31 fungi identified by Dimitrios [37].

### 4.6. Mating-type Locus of A. heimuer

In this study, the genetic linkage map and genomics data of the mating-type locus of *A. heimuer* were combined to locate the mating-type locus and clarify its gene composition. The results show that the two methods could confirm each other, indicating that the results were accurate. A mating-type locus was located on linkage group 8 on scaffold45, which was consistent with the results of the genomics analysis. The distance between marker SSR669 and the mating-type locus on the linkage map was 18.9 cM, and the corresponding distance on scaffold45 was 88.535 kb. By comparing and analyzing the mating-type sequences of different strains A14-8, A14-5,18-119 and Dai 13782, it was found that the mating-type loci of different strains had the same compositions and structures. All of the mating-type structures were composed of two subunits, α and β, but the gap between the two subunits was quite different, from 145 to 2198 bp. The bioinformatics analysis also detected pheromone and pheromone receptor genes in the *A. heimuer* genome. In basidiomycetes with a tetrapolar mating-type system, these genes worked together to control nuclear migration and the fusion of clamp cells with subapical cells [47]. Three hypotheses of the evolutionary relationship between bipolar and tetrapolar mating-system types have been proposed: (1) Factor A and B, which originally regulated mating incompatibility on different chromosomes, are linked by gene conversion or recombination, demonstrating that the mating-type system is bipolar [48,49,50]; (2) Factor A and B are located on different chromosomes and one has lost its function in regulating mating (such as in *Coprinellus disseminates* [51], *P. nameko* [52], and *Phanerochaete chrysosporium* [53]), and although pheromone and receptor genes are present, only transcription factors (*HD* genes) control the mating; and (3) The function of one mating-type gene is replaced by another gene. *A. heimuer* is speculated to be the second type, which means that although the pheromone and pheromone receptor genes are found, they no longer regulate mating incompatibility.4.7. Synteny of A. heimuer on MAT-A

The *mip* and *beta-fg* genes of *A. heimuer* and *A. subglabra* were found to be located on one side of MAT-A, the same as was found for *L. edodes* [54], but the distance between the two genes in *HD*148-2-1 was 51.91 kb, which is much longer than the distance in *L. edodes* (0.76 kb). In the *F. velutipes* [43,54], *C. cinereus* [43,45,54], *L. bicolor* [49,53,54], *Coprinellus disseminatus* [53], *P. djamor* [54], *P. chrysosporium* [53], *P. nameko* [45], *A. bisporus* [45], *V. volvacea* [45], and *Postia placenta* [53] genomes, the *mip* and *beta-fg* genes flanked the mating-type genes. The tight linkage between the *mip* locus and the genes of MAT-A of homobasidiomycetes were reported to be conserved within <1 kb, for example, in the genomes of the model mushroom *C. cinereus* [55] and *S. commune* [56], whereas in *A. heimuer* strains A14-8 and Dai 13782 the distances were 59.5 and 67.6 kb, respectively, which are much longer than those of *C. cinereus* [55] and *S. commune*. The mating-type genes of mushroom-forming fungi are essential for the formation of dikaryotic mycelia and the development of fruiting bodies. The research on the structure and synteny of MAT-A provided the reference for the evolution study of different species in Agaricomycetes.

## 5. Conclusions

The efficient utilization of substrate is one of the key factors in improving mushroom yield. One of the essential questions affecting the utilization of substrates is how the lignocellulose was degraded [57]. Therefore, in-depth studies on the composition and mechanism of CAZyme can lay the groundwork for breeding edible mushrooms that are efficient in the degradation of lignocellulose. On the basis of the *A. heimuer* genome sequence, we analyzed the genes involved in degrading lignocellulolytic enzymes, screening out the candidate genes for the nutritional utilization. The results can be used to develop molecular markers for breeding high-yield cultivars of *A. heimuer*. Bioethanol production is an important topic worldwide due to energy security. This study has identified alcohol dehydrogenase in the *A. heimuer* genome and showed that *A. heimuer* has the potential to produce ethanol as a reactor by consolidated bioprocessing. The mating loci of *A. heimuer* were mapped to a genetic linkage map and the structure and coding sequences of the mating loci were analyzed using genome-wide sequence information. Research on the structure and synteny of MAT-A provided the reference for the evolution study of different species in Agaricomycetes. In sum, these results will contribute to further studies of the genetics, evolution, breeding, bioethanol development and cultivation of *A. heimuer*.

## Figures and Tables

**Table 1:** Features of the *Auricularia heimuer* genome.

General Features.		Properties of Predicted Gene Models	
Genome size (Mb)	43.6	KEGG alignment	2257
GC content (%)	57.09	Interpro signature	9254
Scaffolds N50 (bp)	276,651	Pfam alignment	12,279
Number of scaffolds	535	CAZyme alignment	509
Number of predict gene models	14,094	Signal peptide	1694
Average of gene length (bp)	1737.54	Transmembrane domain	2441
Average of cds length (bp)	1390		
Average of exon number	5.71		
Average of intron number	4.71		
Average of exon length (bp)	244		
Average of intron length (bp)	74		
Repetitive sequences (%)	3.73		

**Table 2:** The number of CAZymes and AAs of *A. heimuer* compared with those of other fungi.

CAZy Family	*A.hei*	*T.fuc*	*A.sub*	*L.edo*	*F.vel*	*P.ost*	*S.com*	*G.luc*	*S.lac*	*C.cin*	*A.bis*	*T.mes*	*V.vol*
CBM	60	11	56	30	22	54	20	28	12	71	29	10	66
CE	18	14	18	11	19	14	12	8	7	23	18	8	11
GH	265	107	292	209	190	212	206	205	125	178	152	56	195
GT	51	53	51	62	57	48	54	52	37	51	41	50	51
PL	11	2	11	3	20	18	11	6	2	6	4	2	19
AA	104	14	124	85	91	133	78	99	41	124	90	8	118
sum	509	201	552	400	399	479	381	398	224	453	334	134	460

CAZy Family: GH, glycoside hydrolase; GT, glycosyltransferase; PL, polysaccharide lyases; CE, carbohydrate esterases; CBM, carbohydrate-binding modules; AA, auxiliary activities. Species abbreviations and genome references: *A.hei* is *A. heimuer*; *A.sub* is *A. subglabra*; *T.fuc* is *T. fuciformis*; *L.bic* is *L. bicolor*; *L.edo* is *Lentinula edodes*; *C.cin* is *C. cinereus*; *F.vel* is *F. velutipes*; *P.ost* is *P. ostreatus*; *S.lac* is *S. lacrymans*; *S.com* is *S. commune*; *A.bis* is *A. bisporus*; *V.vol* is *V. volvacea*; *G.luc* is *G. lucidum*; *T.mes* is *T. mesenterica*.

**Table 3:** Genes numbers of CAZyme families involved in the wood degradation of *A. heimuer.*

Substate	CAZy Family	Species											
		*A. hei.*	*A.sub*	*L. edo*	*F. vel*	*P. ost*	*S. com.*	*S. lac.*	*G. luc*	*A. bis*	*V. vol*	*L. bic*	*C. cin.*
Crystalline cellulose	CBM1	43	43	23	15	31	4	8	20	16	54	1	50
	GH6	2	2	1	2	3	1	1	1	1	4	0	5
	GH7	5	6	3	3	16	2	0	3	1	12	0	6
	AA9	19	20	13	19	28	22	5	15	11	31	3	34
	sum	69	71	40	39	78	29	14	39	29	101	4	95
Lignin	AA1_1	0	0	14	8	11	2	4	13	11	11	10	17
	AA1_2	1	1	1	2	1	0	2	1	1	0	4	0
	AA2	21	21	10	3	9	2	1	10	5	9	3	4
	AA3_1	1	0	0	1	0	0	0	0	1	0	0	1
	AA3_2	22	34	17	20	36	15	7	26	28	26	8	27
	AA3_3	3	6	4	4	4	4	5	4	4	4	2	2
	AA3_4	1	3	1	0	0	1	0	0	0	0	0	0
	AA4	1	1	1	3	1	2	2	1	1	1	0	0
	AA5_1	5	7	5	7	15	2	3	10	8	4	10	6
	AA6	4	4	2	3	2	4	2	2	4	2	2	3
	AA7	15	14	11	19	23	12	8	12	13	26	7	18
	AA8	9	11	2	3	1	1	2	1	5	1	0	3
	sum	83	102	68	73	103	45	36	80	81	84	46	81

CAZy Family: GH, glycoside hydrolase; GT, glycosyltransferase; PL, polysaccharide lyases; CE, carbohydrate esterases; CBM, carbohydrate-binding modules; AA, auxiliary activities. Species abbreviations and genome references: *A.hei* is *A. heimuer*; *A.sub* is *A. subglabra*; *T.fuc* is *T. fuciformis*; *L.bic* is *L. bicolor*; *L.edo* is *Lentinula edodes*; *C.cin* is *C. cinereus*; *F.vel* is *F. velutipes*; *P.ost* is *P. ostreatus*; *S.lac* is *S. lacrymans*; *S.com* is *S. commune*; *A.bis* is *A. bisporus*; *V.vol* is *V. volvacea*; *G.luc* is *G. lucidum*. The background color from dark blue to dark red showed the increased numbers.

**Table 4:** α and β subunit structure of the mating-type locus in the *A. heimuer* genomes.

**Strain**	**Mating-Type Locus**	**Gene**	**Length of Amino Acid (aa)**	**Subunit**	**The Gap in the Same Subunit (bp)**	**The Gap Between α and β Subunit (kb)**
A14-8	scaffold45	*HD1-1*(MN267022)	683	α subunit	153	36.725
		*HD1-2*(MN267023)	626			
A14-5	contig976	*HD1-1*(MN267026)	593	α subunit	294	36.716
		*HD1-2*(MN267027)	683			
18-119	contig313	*HD1-1*(MN267028)	684	α subunit	1143	37.563
		*HD1-2*(MN267029)	633			
Dai 13782	NEKD01000007.1	*HD1-1*(MN442077)	615	α subunit	171	41.16
		*HD1-2*(MN442078)	613			
**Strain**	**Mating-type Locus**	**Gene**	**Length of Amino Acid (aa)**	**Subunit**	**The Gap in the Same Subunit (bp)**	
A14-8	scaffold45	*HD2-1*(MN267021)	693	β subunit	2198	
		*HD2-2*(MN267024)	628			
A14-5	contig976	*HD2-1*(MN267030)	628	β subunit	153	
		*HD2-2*(MN267031)	626			
18-119	contig313	*HD2-1*(MN267032)	622	β subunit	411	
		*HD2-2*(MN267033)	650			
Dai 13782	NEKD01000007.1	*HD2-1*(MN442079)	715	β subunit	145	
		*HD2-2*(MN442080)	599			

## Supplementary Materials

The following are available online at https://www.mdpi.com/2309-608X/6/1/37/s1.
